# *Staphylococcus aureus* small colony variants are susceptible to light activated antimicrobial agents

**DOI:** 10.1186/1471-2180-13-201

**Published:** 2013-09-06

**Authors:** Sarah Tubby, Michael Wilson, John A Wright, Ping Zhang, Sean P Nair

**Affiliations:** 1 Department of Microbial Diseases, UCL Eastman Dental Institute, University College London, 256 Gray’s Inn Road, London WC1X 8LD, UK; 2 Current address: Department of Veterinary Medicine, University of Cambridge, Madingley Road, Cambridge CB3 0ES, UK

**Keywords:** *Staphylococcus aureus*, Lethal photosensitisation, Photodynamic therapy, Small colony variant

## Abstract

**Background:**

Antibiotic therapy can select for small colony variants of *Staphylococcus aureus* that are more resistant to antibiotics and can result in persistent infections, necessitating the development of more effective antimicrobial strategies to combat small colony variant infections. Photodynamic therapy is an alternative treatment approach which utilises light in combination with a light-activated antimicrobial agent to kill bacteria via a non-specific mechanism of action. In this study, we investigated whether the combination of 665 nm laser light and the light-activated antimicrobial agent methylene blue was able to successfully kill *S. aureus* small colony variants. *S. aureus* and isogenic stable small colony variant were exposed to varying doses (1.93 to 9.65 J/cm^2^) of 665 nm laser light in the presence of varying concentrations (1 to 20 μM) of methylene blue.

**Results:**

The combination of 665 nm laser light and methylene blue was found to be an effective strategy for the killing of small colony variants. At the highest light dose (9.65 J/cm^2^) and methylene blue concentration (20 μM) tested, the number of viable bacteria decreased by approximately 6.9 log_10_ for the wild type and approximately 5 log_10_ for the small colony variant.

**Conclusions:**

These results suggest that photodynamic therapy has potential for use in the treatment of superficial infections caused by small colony variants of *S. aureus* and supports further research in this field.

## Background

Small colony variants (SCVs) of *Staphylococcus aureus* are a naturally-occurring subpopulation often associated with chronic antibiotic exposure [1]. *S. aureus* SCVs are characterized by their slow growth rate and small colony size relative to the parent strain, and can cause persistent infections in the lungs of cystic fibrosis patients and infections of skin, bone and implanted devices [2].

*S. aureus* SCVs are clinically important due to their reduced susceptibility to antibiotics. SCVs are commonly auxotrophs for hemin, menadione or thymidine, resulting in electron transport chain defects and consequently reduced membrane potential and reduced uptake of cationic antibiotics [3]. Resistance to cell wall–active antibiotics such as β-lactams occurs due to the slow growth rate and reduced cell wall metabolism of SCVs [4].

Given their persistent nature and their selection by and resistance to conventional antibiotics, there is a need to identify effective therapies for SCV infections. One potential novel strategy is photodynamic therapy, which utilizes light in combination with a light-activated antimicrobial agent, known as a photosensitiser, to generate toxic reactive oxygen species such as free radicals and singlet oxygen. Upon irradiation, the photosensitiser undergoes a transition from a low energy ground state to a higher energy triplet state, which can then react with biomolecules to produce free radicals or with molecular oxygen to produce highly reactive singlet oxygen. These reactive oxygen species can oxidise many biological structures and kill bacteria via several mechanisms, most notably by damaging the cytoplasmic membrane [5].

There are several potential advantages of light-activated antimicrobial agents over conventional antimicrobial therapy. Firstly, collateral damage to the host or host microbiota is limited due to the very short half-life and diffusion distance of the reactive oxygen species produced. Secondly, resistance is unlikely as reactive oxygen species kill bacteria through non-specific mechanisms, by attacking proteins, lipids and nucleic acids. We have previously shown that light-activated antimicrobial agents such as methylene blue and tin (IV) chlorin e6 are effective against meticillin-sensitive *S. aureus*, epidemic meticillin-resistant *S. aureus* (MRSA), community-acquired MRSA and vancomycin intermediate *S. aureus* (VISA) [6,7], and are effective for decolonizing wound infections *in vivo*[8]. However given that most light activated antimicrobials are cationic compounds and that SCVs have a reduced membrane potential which makes them more resistant to cationic antimicrobials it is possible that such light activated antimicrobials would not be effective at killing SCVs. Therefore the aim of this study was to determine the capacity of the cationic light activated antimicrobial agent methylene blue in combination with 665 nm laser light to kill *S. aureus* SCVs.

## Results and discussion

As mentioned small colony variants of *S. aureus* have been reported to have increased resistance to conventional antimicrobials such as aminoglycosides. In this study we determined that the minimum inhibitory concentration of the aminoglycoside kanamycin against the *hemB* and *menD* small colony variants was 8-fold higher (128 μg/ml) than the isogenic parent strains (16 μg/ml).

**Figure 1:**
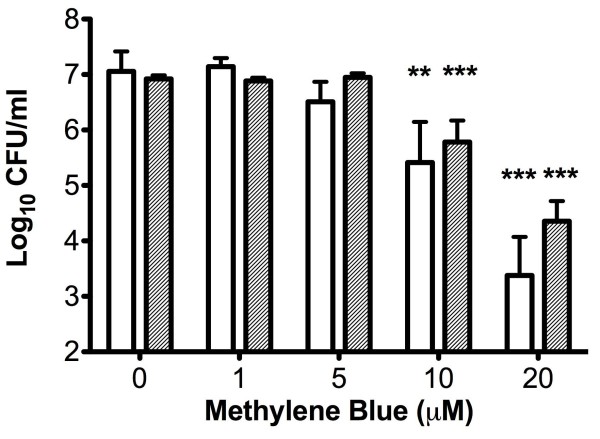
**Number of viable bacteria recovered following exposure to 1.93 J/cm**^**2 **^**of 665 nm laser light and different concentrations of methylene blue.** The clear bars represent recovery of the wild type strain LS-1 and the grey bars the isogenic *hemB* SCV. Error bars represent the standard deviation from the mean. **P < 0.01, ***P < 0.001 (ANOVA).

**Figure 2:**
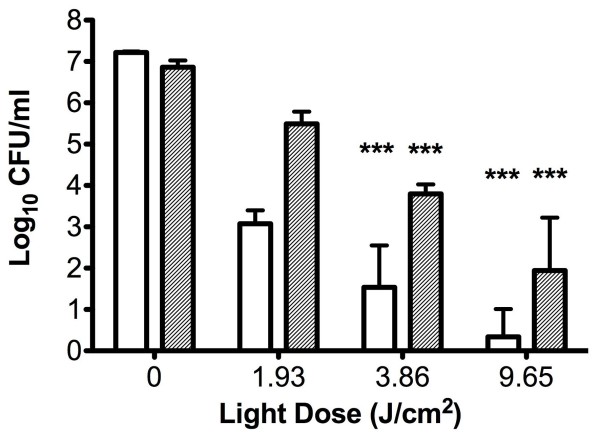
**Number of viable bacteria recovered following exposure to methylene blue and different doses of 665 nm laser light.** The clear bars represent recovery of the wild type strain LS-1 and the grey bars the isogenic *hemB* SCV. Error bars represent the standard deviation from the mean. ***P < 0.001 (ANOVA).

The *hemB* SCV and its isogenic parent were both found to be susceptible to photodynamic killing using methylene blue and 1.93 J/cm^2^ of 665 nm laser light in a methylene blue concentration-dependent manner (Figure 1). Neither laser light nor photosensitiser alone had any effect on bacterial viability (data not shown). The *menD* SCV and its wild-type parent were also susceptible to photodynamic killing by methylene blue (20 μM) and 1.93 J/cm^2^ of 665 nm laser light, with reductions in cell viability of 3.5 log_10_ and 4.1 log_10_, respectively (data not shown). Increasing the light dose was found to significantly increase the killing of both the *hemB* SCV and its parent strain; the highest light dose examined (9.65 J/cm^2^) resulted in reductions in viable cells of approximately 6.9 log_10_ and 5 log_10_ respectively (Figure 2). There was no significant difference between the kills observed for both strains when a light dose of 9.65 J/cm^2^ was used for the experiments.

Small colony variants of *S. aureus* represent a serious challenge to clinicians treating infections caused by these microorganisms [2] due to the increased antibiotic resistance and persistent infections that are characteristic of SCVs [1,3,4]. It would therefore be advantageous to develop a therapeutic strategy with a differing mode of action to those antibiotics for which lower susceptibility is observed. We have previously shown that light-activated antimicrobial agents, which have a non-specific mode of action, are highly effective at killing *S. aureus*[6-8]. To investigate the capacity of the light-activated antimicrobial agent methylene blue in combination with laser light for eradicating SCVs of *S. aureus*, we used stable hemin (*hemB* mutant) and menadione (*menD* mutant) auxotrophic SCVs of *S. aureus*, which we found to have an 8-fold increase in resistance to the aminoglycoside kanamycin. Our results demonstrate that the combination of methylene blue and laser light of 665 nm effectively kills *S. aureus* SCVs, suggesting that photodynamic therapy could be a promising alternative therapy for SCV infections. Selection for SCVs and development of resistance are unlikely due to the non-specific mechanism of action of photodynamic therapy, representing an advantage over conventional antibiotic treatment. One potential limitation to the effectiveness of photodynamic therapy is that in some infections SCVs enter the cytoplasm of host cells [3]. In such cases it is likely that higher photodynamic therapy doses would be required resulting in some collateral damage to host tissue. One possible way to overcome this problem would be to develop photosensitisers that target the intracellular bacteria specifically.

Photodynamic therapy has been proposed for the decontamination of the anterior nares in cases of MRSA carriage [6,8,9]. Cases of infections associated with concomitant colonisation of the anterior nares by *S. aureus* small colony variants have been reported in the literature [10-12]; therefore, photodynamic therapy may also be of use as a decontamination strategy in cases where the anterior nares represent a reservoir of SCVs.

## Conclusion

In conclusion, we propose that photodynamic therapy has potential for use in the treatment of superficial infections by SCVs of *S. aureus* and for nasal decolonisation.

## Methods

The *S. aureus* strains used were the laboratory strain 8325–4 and an isogenic mutant, D1324, disrupted in *menD*[13], (a gift from Professor Richard Proctor), and LS-1 and its isogenic mutant disrupted in *hemB*[14]. *S. aureus* was maintained by subculture on blood agar (Oxoid Ltd, UK) incubated aerobically at 37°C. For experimental purposes, bacteria were inoculated into Brain Heart Infusion broth and cultured aerobically for 16 hrs at 37°C, with shaking at 200 rpm. Cultures were then centrifuged and resuspended in an equal volume of PBS and the optical density adjusted to 0.05 at 600 nm, corresponding to approximately 1 × 10^7^ colony forming units (CFU) per mL. Methylene blue (C16H18ClN3S.3H2O) and all other reagents were purchased from Sigma-Aldrich (UK).

The MIC of kanamycin was determined according to the CLSI microbroth dilution. A Periowave™ diode laser (Ondine Biomedical Inc., Canada), which emits light with a wavelength of 665 nm was used throughout the study. The power output of the laser was 73 mW and the beam diameter was 1.7 cm. The laser system was set up so that the laser beam covered the entire well of a microtitre plate in which experiments were performed. To examine the effect of photosensitiser concentration on the photodynamic killing of *S. aureus* SCVs, methylene blue was diluted in PBS to give final concentrations of 1, 5, 10 and 20 μM. Methylene blue (50 μL) was added to an equal volume of the inoculum in triplicate wells of a sterile, flat-bottomed 96-well plate and irradiated with 665 nm laser light with an energy density of 1.93 J/cm^2^, with stirring. Three additional wells containing 50 μL of methylene blue and 50 μL of the bacterial suspension were kept in the dark to assess the toxicity of the photosensitiser alone. To assess the toxicity of laser light alone, 50 μL PBS was added to 50 μL of the inoculum in a further six wells, three of which were irradiated with laser light and the remaining three kept in the dark. Following irradiation/dark incubation, samples were serially diluted 10-fold in PBS and plated onto 5% horse blood agar plates in triplicate. The plates were incubated aerobically overnight at 37°C, following which the surviving CFU/mL were enumerated by viable counting. Experiments were performed three times in triplicate.

To examine the effect of laser light dose on the photodynamic killing of the SCVs, methylene blue was diluted in PBS to give a final concentration of 20 μM. Experiments were performed as described above, but bacteria were irradiated with 1.93 J/cm^2^, 3.86 J/cm^2^ or 9.65 J/cm^2^ of 665 nm laser light, with stirring. Following irradiation/dark incubation, viable bacteria were enumerated as described as above.

## Competing interests

ST received a studentship stipend from Ondine Biopharma Inc. and MW holds shares in Ondine Biopharma Inc.

## Authors’ contributions

ST: participated in the study design, carried out the experimental work, performed the statistical analysis and drafted the manuscript. MW: conceived of the study, participated in its design and helped to draft the manuscript. JAW carried out the experimental work and helped draft the manuscript. PZ carried out the experimental work and helped draft the manuscript. SPN: conceived of the study, participated in its design, interpreted the data, and drafted the manuscript. All authors read and approved the final manuscript.
